# Infective Aortic Valve Endocarditis in a Patient With Mixed Connective Tissue Disease

**DOI:** 10.7759/cureus.20681

**Published:** 2021-12-25

**Authors:** Leonor Boavida, Joana Azevedo Carvalho, Frederico Batista, Susana Oliveira, José Alves

**Affiliations:** 1 Department of Internal Medicine IV, Hospital Professor Doutor Fernando Fonseca, Lisbon, PRT; 2 Department of Internal Medicine IV, Hospital Professor Doutor Fernando Fonseca, Amadora, PRT

**Keywords:** enterococcus faecalis, autoimmunity, mixed connective tissue disease, infective endocarditis relapse, immunosuppression

## Abstract

Mixed connective tissue disease (MCTD) is a rare autoimmune disorder that is characterized by overlapping clinical features of systemic lupus erythematosus (SLE), scleroderma, and myositis. Both SLE and mixed connective tissue disease patients are more prone to have acute endocarditis, and immunosuppression is a risk factor for recurrence of infective endocarditis.

We present the case of a 53-year-old female with mixed connective tissue disease presenting with interstitial lung disease and precapillary pulmonary hypertension. The patient was chronically medicated with prednisolone, mycophenolate mofetil, and hydroxychloroquine. She was admitted for *Enterococcus faecalis *infective endocarditis and was treated with a four-week course of ceftriaxone and ampicillin. Immunosuppressive chronic medication was maintained due to severe lung involvement. One month later, the patient was re-admitted due to respiratory infection with identification of influenza B virus. However, fever persisted for over one week, and subsequent relapse of the *E. faecalis* infective endocarditis was found. The diagnosis was made based on blood cultures and a transoesophageal echocardiogram. No other focus of infection was identified. She completed a six-week course of vancomycin and gentamicin and underwent cardiac surgery with success. This case highlights the difficulty of the management of immunosuppressed patients in the presence of serious infections.

## Introduction

Mixed connective tissue disease (MCTD) is a rare autoimmune disorder that is characterized by overlapping clinical features of systemic lupus erythematosus (SLE), scleroderma, and myositis, with the presence of a distinctive antibody against U1-ribonucleoprotein (RNP) [1].

Systemic lupus erythematosus and mixed connective tissue disease patients are more prone to have acute endocarditis, and immunosuppression is a risk factor for the recurrence of infective endocarditis [2,3]. We present a case of infective endocarditis in an immunocompromised patient with MCTD and severe lung involvement that warranted maintenance of immunosuppressive drugs during antibiotic treatment. This may have contributed to the infective endocarditis relapse one month later culminating in a longer course of treatment and need for surgical valve repair.

This case was previously presented as a poster at the 22nd Annual Congress of the European Association of Cardiovascular Imaging (EACVI) in December 2018.

## Case presentation

The patient was a 53-year-old female with MCTD with severe interstitial lung disease and precapillary pulmonary hypertension (PH) (requiring continuous administration of 3 L/min of oxygen via nasal cannula), myositis, pericardium, and cutaneous involvement. She had positive antinuclear and anti-U1 ribonucleoprotein antibodies. She was on daily prednisolone (PDN) 5 mg, mycophenolate mofetil (MMF) 1.5 g, and hydroxychloroquine 400 mg. The patient was admitted to the hospital due to fever and worsening of dyspnea with minimal exertion over the previous four weeks. Physical examination revealed a temperature of 38.4ºC, respiratory rate of 20 breaths/minute, blood pressure of 110/50 mmHg, oxygen saturation value of 89% on 3 L/min of oxygen via nasal cannula, bilateral lung crackles, a grade III diastolic murmur at the aortic area, and symmetrical lower limb grade 2 pitting edema.

**Figure 1:**
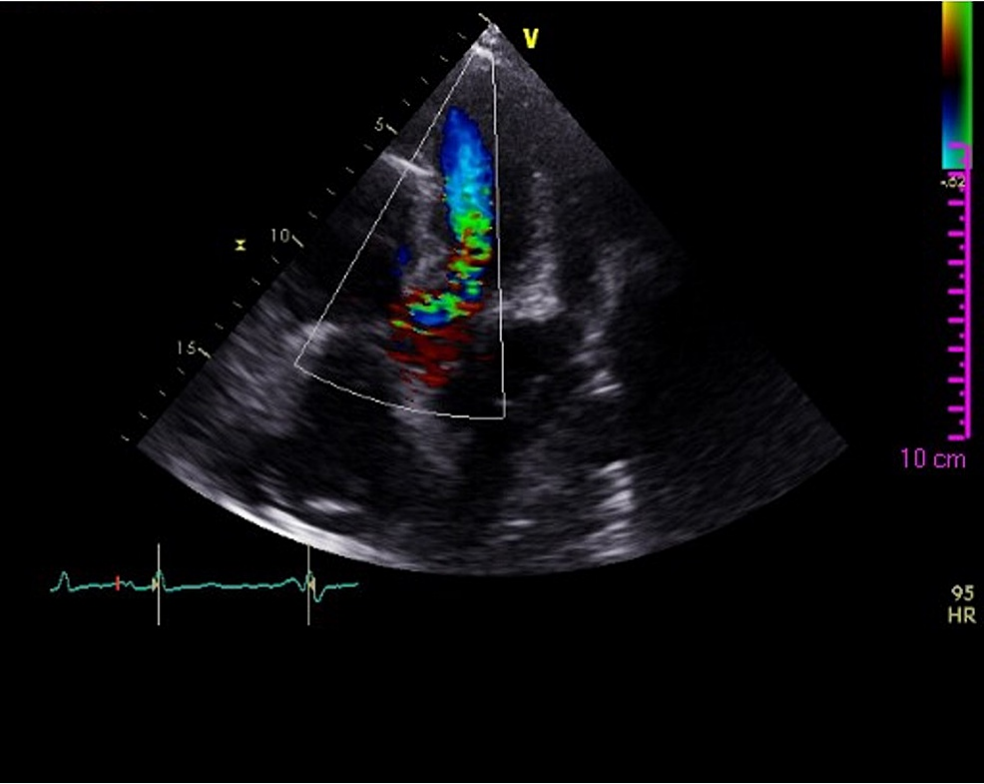
Transthoracic echocardiogram color-Doppler image of moderate to severe aortic regurgitation

**Figure 2:**
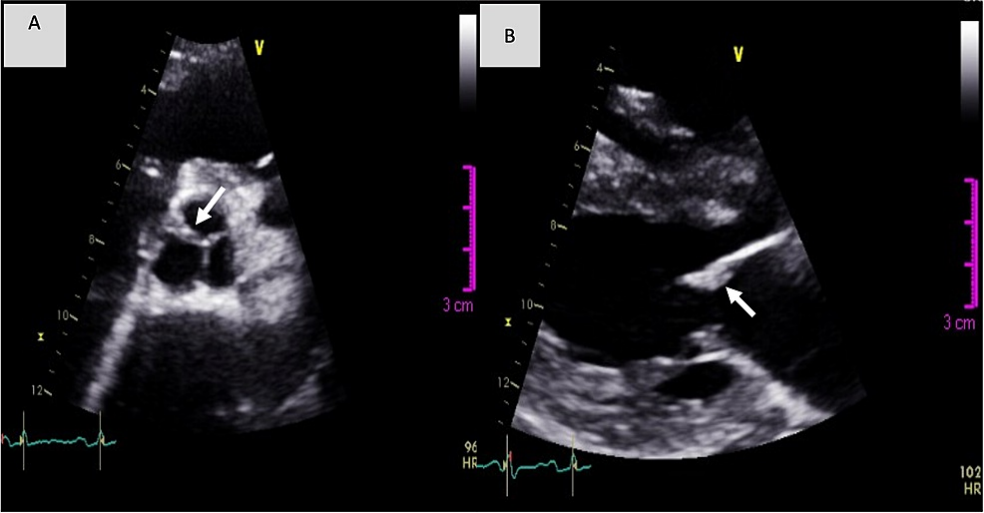
(A) Small vegetations on the aortic valve and (B) in the anterior mitral leaflet in transthoracic echocardiogram (arrows)

Blood tests showed a high leukocyte count (13,200 µ/L) and an elevated C-reactive protein (CRP) (12 mg/dL). Two sets of blood cultures were positive for Enterococcus faecalis (EF) with antibiotic susceptibility to ampicillin, gentamycin, and vancomycin. Transthoracic echocardiogram (TTE) revealed a preserved left ventricular ejection fraction (LVEF) of 62% but depicted a new onset of moderate to severe aortic regurgitation (Figure 1) with left aortic cusp and anterior mitral leaflet thickening (Figure 2) suggesting small vegetations while confirming signs of PH. Transesophageal echocardiogram (TEE) confirmed two small vegetations on the aortic valve with a compromised aortic cusp coaptation and another two vegetations on the anterior mitral leaflet with mild mitral regurgitation. There were no signs of abscess or fistulae. The diagnosis of infective endocarditis (IE) was made, and the patient completed four weeks of ceftriaxone and ampicillin, with negative blood cultures at discharge, alongside diuretic therapy, with symptomatic relief. Because of the severity of her underlining disease, particularly her severe lung involvement, PDN and MMF were maintained throughout the antibiotic treatment.

One month later, the patient was re-admitted due to fever, dry cough, and myalgia. On clinical examination, she was normotensive, febrile with a temperature of 38.7ºC, and had an oxygen saturation value of 85% on 4 L/min of oxygen via nasal cannula. She was positive for the influenza B virus. Still, fever as well as high CRP and leukocyte count persisted over one week, and two sets of blood cultures were again positive for EF with the same antibiotic sensitivity as the isolate from the previous hospitalization. There was no other obvious local infection, and body CT showed no signs of infection or abscesses.

**Figure 3:**
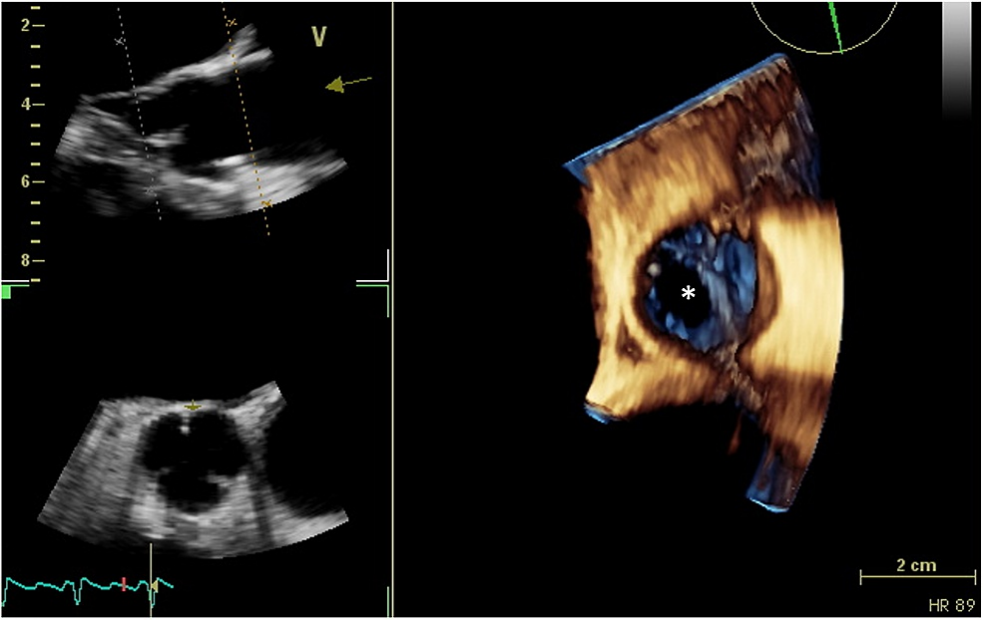
Vegetations of the aortic valve cusps with non-coronary cusp perforation (asterisk) in transesophageal echocardiogram

TEE was repeated and revealed multiple vegetations of the aortic valve cusps with prolapse and perforation of the non-coronary cusp and severe aortic regurgitation (Figure 3) as well as several vegetations on the mitral valve leaflets with moderate mitral regurgitation, without significant left ventricular function compromise (LVEF of 58%). No abscesses were seen. The tricuspid valve had no vegetations, but moderate tricuspid regurgitation was seen with a pulmonary artery systolic pressure (PASP) of 108 mmHg. The diagnosis of relapsing IE was made, and the patient completed a six-week course of vancomycin and gentamicin. As she remained in class IV New York Heart Association (NYHA), she was offered double-valve surgery with mitral valve repair and aortic valve replacement with a mechanical valve. The procedure was successful, and she was discharged two weeks later in class II NYHA, under treatment with warfarin, beta-blocker, and diuretic therapy as well as with phosphodiesterase 5 inhibitor and endothelin receptor antagonist for the management of PH.

## Discussion

Patients with MCTD may have clinical features of SLE, systemic sclerosis, and polymyositis and are treated with immunosuppressive therapy according to their clinical manifestations [1]. One possible cardiac manifestation is Libman-Sacks endocarditis (LSE), which can affect 10% of patients with SLE [2]. Nevertheless, SLE patients are at a higher risk of developing IE, particularly when treated with steroids or if they have valve abnormalities [3]. In patients with SLE or MCTD, it is critical to distinguish between LSE and IE because management is distinct.

LSE is characterized by sterile vegetations that usually form on the ventricular surface of the mitral valve but can affect any valve. It is typically associated with normal inflammatory markers and is more often present in patients with antiphospholipid syndrome [4,5]. The prevalence of LSE in patients with SLE is 4%-6%, although it can be as high as 40% in autopsies [6]. Recommended treatment is anticoagulation, and, if involving large vegetation, surgical repair may be considered [5,7].

Contrastingly, IE patients have bacteremia, fever, high CRP and leukocyte count, and positive blood cultures for the infective agent (provided that they are collected as recommended, including for agents of the *Haemophilus *species, *Aggregatibacter *species, *Cardiobacterium hominis*, *Eikenella corrodens*, and *Kingella *species [HACEK] group) [8,9], as seen in our case.

After having completed a course of antibiotic treatment, our patient presented with an IE relapse. IE relapse occurs when the same microorganism is causing a new onset of IE. It suggests a failure to eradicate a focal nidus of bacteria [10] due to immunosuppression or therapy failure [11]. IE relapse is distinctive from reinfection as the latter is not related to the first IE episode and may be caused by a new microorganism [11]. Our patient maintained the treatment with PDN and MMF due to severe lung disease, requiring oxygen therapy, which may have contributed to an IE relapse. After IE relapse, both PDN and MMF doses were reduced, and antibiotic treatment was altered to vancomycin and gentamicin through a longer course of treatment (six weeks). The choice of antibiotic relied on vancomycin as it is associated with a more successful reduction of vegetation size and a faster treatment of Gram-positive bacteria, compared to cephalosporines [12].

Since the patient was gravely symptomatic due to severe PH, severe aortic regurgitation (due to IE leaflets destruction), and moderate mitral regurgitation, in class IV NYHA, she underwent aortic and mitral valve repair after IE treatment, which provided symptomatic benefit (now in class II NYHA).

## Conclusions

This case highlights the difficulty of managing immunocompromised patients in a situation of serious infection and the importance of evaluating the risks and benefits of maintaining immunosuppressive therapy. In this case, immunosuppression probably contributed to the infective endocarditis relapse warranting a longer course of antibiotic treatment and surgical valve repair.
